# Vitamin D status and risk of incident tuberculosis disease: A nested case-control study, systematic review, and individual-participant data meta-analysis

**DOI:** 10.1371/journal.pmed.1002907

**Published:** 2019-09-11

**Authors:** Omowunmi Aibana, Chuan-Chin Huang, Said Aboud, Alberto Arnedo-Pena, Mercedes C. Becerra, Juan Bautista Bellido-Blasco, Ramesh Bhosale, Roger Calderon, Silvia Chiang, Carmen Contreras, Ganmaa Davaasambuu, Wafaie W. Fawzi, Molly F. Franke, Jerome T. Galea, Daniel Garcia-Ferrer, Maria Gil-Fortuño, Barbará Gomila-Sard, Amita Gupta, Nikhil Gupte, Rabia Hussain, Jesus Iborra-Millet, Najeeha T. Iqbal, Jose Vicente Juan-Cerdán, Aarti Kinikar, Leonid Lecca, Vidya Mave, Noemi Meseguer-Ferrer, Grace Montepiedra, Ferdinand M. Mugusi, Olumuyiwa A. Owolabi, Julie Parsonnet, Freddy Roach-Poblete, Maria Angeles Romeu-García, Stephen A. Spector, Christopher R. Sudfeld, Mark W. Tenforde, Toyin O. Togun, Rosa Yataco, Zibiao Zhang, Megan B. Murray

**Affiliations:** 1 Department of Internal Medicine, McGovern Medical School at the University of Texas Health Science Center, Houston, Texas, United States of America; 2 Department of Global Health and Social Medicine, Harvard Medical School, Boston, Massachusetts, United States of America; 3 Department of Microbiology and Immunology, Muhimbili University of Health and Allied Sciences, Upanga West, Dar es Salaam, Tanzania; 4 Epidemiology Division, Public Health Center, Castellon, Spain; 5 Department of Obstetrics & Gynecology, Byramjee Jeejeebhoy Government Medical College, Pune, India; 6 Partners in Health—Socios En Salud Sucursal, Lima, Peru; 7 Department of Pediatrics, Alpert Medical School of Brown University, Providence, Rhode Island, United States of America; 8 Channing Division of Network Medicine, Department of Medicine, Brigham and Women's Hospital, Harvard Medical School, Boston, Massachusetts, United States of America; 9 Department of Global Health and Population, Harvard T.H. Chan School of Public Health, Boston, Massachusetts, United States of America; 10 School of Social Work, University of South Florida, Tampa, Florida, United States of America; 11 Biochemical Laboratory, Hospital General, Castellon, Spain; 12 Microbiology Laboratory, Hospital General, Castellon, Spain; 13 Division of Infectious Diseases, Department of Medicine, Johns Hopkins University School of Medicine, Baltimore, Maryland, United States of America; 14 Byramjee Jeejeebhoy Government Medical College-Johns Hopkins University CRS, Pune, India; 15 Department of Pathology and Microbiology, Aga Khan University, Karachi, Pakistan; 16 Department of Pediatrics and Child Health and Biological and Biomedical Sciences, Aga Khan University, Karachi, Pakistan; 17 Department of Pediatrics, Byramjee Jeejeebhoy Government Medical College, Pune, India; 18 Center for Biostatistics in AIDS Research and Department of Biostatistics, Harvard T.H. Chan School of Public Health, Boston, Massachusetts, United States of America; 19 Department of Internal Medicine, Muhimbili University of Health and Allied Sciences, Upanga West, Dar es Salaam, Tanzania; 20 Medical Research Council Gambia at London School of Hygiene and Tropical Medicine, Banjul, The Gambia; 21 Departments of Medicine and of Health Research and Policy, Stanford University School of Medicine, Stanford, California, United States of America; 22 Laboratory Hospital Regional Antofagasta, Antofagasta, Chile; 23 Division of Infectious Diseases, Department of Pediatrics, University of California San Diego, La Jolla, California, United States of America; 24 Division of Allergy and Infectious Diseases, Department of Medicine, University of Washington School of Medicine, Seattle, Washington, United States of America; 25 Department of Epidemiology and Biostatistics, McGill University, Montreal, Quebec, Canada; 26 Division of Global Health Equity, Brigham and Women’s Hospital, Harvard Medical School, Boston, Massachusetts, United States of America; Liverpool School of Tropical Medicine, UNITED KINGDOM

## Abstract

**Background:**

Few studies have evaluated the association between preexisting vitamin D deficiency and incident tuberculosis (TB). We assessed the impact of baseline vitamins D levels on TB disease risk.

**Methods and findings:**

We assessed the association between baseline vitamin D and incident TB in a prospective cohort of 6,751 HIV-negative household contacts of TB patients enrolled between September 1, 2009, and August 29, 2012, in Lima, Peru. We screened for TB disease at 2, 6, and 12 months after enrollment. We defined cases as household contacts who developed TB disease at least 15 days after enrollment of the index patient. For each case, we randomly selected four controls from among contacts who did not develop TB disease, matching on gender and year of age. We also conducted a one-stage individual-participant data (IPD) meta-analysis searching PubMed and Embase to identify prospective studies of vitamin D and TB disease until June 8, 2019. We included studies that assessed vitamin D before TB diagnosis. In the primary analysis, we defined vitamin D deficiency as 25–(OH)D < 50 nmol/L, insufficiency as 50–75 nmol/L, and sufficiency as >75nmol/L. We estimated the association between baseline vitamin D status and incident TB using conditional logistic regression in the Lima cohort and generalized linear mixed models in the meta-analysis. We further defined severe vitamin D deficiency as 25–(OH)D < 25 nmol/L and performed stratified analyses by HIV status in the IPD meta-analysis. In the Lima cohort, we analyzed 180 cases and 709 matched controls. The adjusted odds ratio (aOR) for TB risk among participants with baseline vitamin D deficiency compared to sufficient vitamin D was 1.63 (95% CI 0.75–3.52; *p* = 0.22). We included seven published studies in the meta-analysis and analyzed 3,544 participants. In the pooled analysis, the aOR was 1.48 (95% CI 1.04–2.10; *p* = 0.03). The aOR for severe vitamin D deficiency was 2.05 (95% CI 0.87–4.87; *p* trend for decreasing 25–(OH)D levels from sufficient vitamin D to severe deficiency = 0.02). Among 1,576 HIV-positive patients, vitamin D deficiency conferred a 2-fold (aOR 2.18, 95% CI 1.22–3.90; *p* = 0.01) increased risk of TB, and the aOR for severe vitamin D deficiency compared to sufficient vitamin D was 4.28 (95% CI 0.85–21.45; *p* = 0.08). Our Lima cohort study is limited by the short duration of follow-up, and the IPD meta-analysis is limited by the number of possible confounding covariates available across all studies.

**Conclusion:**

Our findings suggest vitamin D predicts TB disease risk in a dose-dependent manner and that the risk of TB disease is highest among HIV-positive individuals with severe vitamin D deficiency. Randomized control trials are needed to evaluate the possible role of vitamin D supplementation on reducing TB disease risk.

## Abstract 2

Megan B. Murray and colleagues combine data from a case-control study in Lima, Peru, with previously published prospective studies to investigate whether low vitamin D is associated with risk of developing tuberculosis disease.

## Author summary

**Why was this study done?:**

- Although multiple lines of evidence suggest that vitamin D may play a role in host susceptibility to tuberculosis (TB) disease, the impact of low vitamin D on the risk of developing TB disease has not yet been firmly established.

**What did the researchers do and find?:**

- We conducted a study in Lima, Peru, in which we measured serum 25–(OH)D levels in individuals at high risk for TB disease and followed them for development of TB over 1 year.
- We pooled individual-level data from seven previously published prospective studies conducted worldwide and from our Lima cohort.
- We found that individuals with low levels of vitamin D were at higher risk of future progression to TB disease.

**What do these findings mean?:**

- These findings suggest that vitamin D deficiency is a risk factor for developing TB disease.
- Randomized control trials are needed to determine whether vitamin D supplementation reduces risk of TB disease.

## Introduction

The global burden of tuberculosis (TB) remains high, with approximately one-fourth to one-third of the world’s population infected with *Mycobacterium tuberculosis* (MTB), and the World Health Organization (WHO) estimates 10 million people developed TB disease in 2017 [1]. Concurrently, vitamin D deficiency (VDD) is a widespread problem globally, with a high degree of geographic variability; reported adult prevalence of VDD ranges from 10% in North America to >80% in parts of Asia [2,3]. Vitamin D is an important regulator of the immune system [4], and in vitro studies have elucidated multiple mechanisms by which vitamin D influences the pathogenesis of TB infection or disease [5–8]. At the level of the macrophage, vitamin D is involved in cathelicidin- and interferon gamma (IFN-γ)-mediated activity against mycobacteria, [5,6], induction of oxidative species [7], and the promotion of phagolysosome fusion, which leads to degradation of mycobacteria [8]. Discoveries about the various ways vitamin D modulates specific host immune responses to TB infection have focused attention on the possibility that low vitamin D levels may contribute to TB disease progression.

Numerous observational studies have also documented lower serum vitamin D levels among TB patients compared to healthy controls, and prior meta-analyses investigating the association between vitamin D and TB have concluded that low vitamin D increases TB disease risk [9–12]. However, most studies were cross-sectional studies and assessed vitamin D status after the diagnosis of active TB disease, rather than the impact of preexisting vitamin D levels on the risk of progression to TB disease. Given TB disease can induce profound metabolic abnormalities, it is unclear whether VDD increases TB disease risk or whether underlying TB infection or disease leads to decreased serum 25–(OH)D levels. Furthermore, prior studies evaluating the association between vitamin D and TB disease have used different cutoffs to categorize vitamin D levels or define VDD [9–12]. Hence, it is challenging to determine whether there is a vitamin D threshold below which individuals are at the greatest risk of TB disease.

Here, we address the association of vitamin D status on the risk of TB progression in two ways. We first report results of a case-control analysis nested in a prospective cohort study of household contacts (HHCs) of TB patients that we conducted in Lima, Peru. We next pool these data with those from other published prospective studies of vitamin D status and TB risk to conduct an individual-participant data (IPD) meta-analysis synthesizing available evidence on the association between vitamin D status and incident TB disease.

## Methods

### Lima cohort study

#### Ethics statement

The Lima cohort study was approved by the Institutional Review Board of Harvard School of Public Health and the Research Ethics Committee of the National Institute of Health of Peru. All study participants or guardians provided written informed consent.

#### Study setting and population

We enrolled a prospective longitudinal cohort of HHCs of index TB patients in Lima, Peru, between September 1, 2009, and August 29, 2012, for a parent study that was designed to identify various host risk factors for TB infection and disease after exposure. We performed secondary analyses nested in this longitudinal cohort evaluating nutritional determinants of TB disease; we did not follow a prespecified protocol for these secondary analyses of nutritional risk factors. We provide a STROBE checklist of items specific to case-control analyses (S1 Text). Details of the nested study design and methods are described elsewhere [13,14]. At the time of diagnosis of an index patient, HHCs were assessed for symptoms of TB (fever, night sweats, weight loss, cough, malaise) and underwent a brief physical exam (lung auscultation, assessment for lymphadenopathy and signs of recent weight loss) conducted by a clinician to rule out TB disease. We referred those with any signs or symptoms of TB for diagnostic evaluation with chest radiograph as well as sputum smear microscopy and mycobacterial culture according to Peru’s national guidelines [15]. Among HHCs without a prior history of TB infection or disease, we assessed baseline TB infection status with the tuberculin skin test (TST). We used a structured questionnaire to obtain clinical, sociodemographic, and environmental information from HHCs. We offered all HHCs HIV testing. We also invited HHCs to provide a baseline blood sample; 60% of HHCs aged 10 years and older complied. According to national guidelines [15], local healthcare staff offered isoniazid preventive therapy (IPT) to all children under 5 years of age, to TST-positive children between ages 5 and 19 years, and to adults with specified comorbidities (HIV, malignancies, immune deficiencies).

We visited households and reevaluated all HHCs for pulmonary and extrapulmonary TB disease at 2, 6, and 12 months after enrollment. We classified HHCs as having incident secondary TB disease if they were diagnosed at least 15 days after index case enrollment and co-prevalent TB disease if they were diagnosed earlier.

At the completion of follow-up, we identified “cases” from among the HHC cohort; these were HIV-negative HHCs with blood samples who developed incident secondary TB disease within 1 year of follow-up. For each case, we randomly selected four controls from among HHCs who were not diagnosed with TB disease during the study period, matching on gender and age by year.

#### Laboratory methods

We stored all blood samples at −80°C from enrollment until end of follow-up. All samples were handled identically, and laboratory personnel were not aware of the case or control status of specimens. Levels of total 25–(OH)D were measured with a commercial competitive enzyme immunoassay kit (Immunodiagnostic Systems, Fountain Hills, AZ, United States), which is sensitive to 5.0 nmol/L. The interassay coefficient of variation for 25–(OH)D ranged from 4.6% to 8.7%. We also measured retinol levels using the high-performance liquid chromatography (HPLC) method described by El-Sohemy and colleagues [16]. Since testing of all blood samples occurred after the end of study follow-up, participants were not diagnosed with VDD during study period and were not offered supplementation.

#### Statistical analysis

We defined VDD as serum 25–(OH)D < 50 nmol/L, vitamin D insufficiency (VDI) as 50–75 nmol/L, and sufficiency as >75 nmol/L [17]. We defined vitamin A deficiency (VAD) as serum retinol < 200 μg/L [18]. We classified adults ≥ 20 years old as underweight (body mass index [BMI] < 18.5 kg/m^2^), normal weight (BMI 18.5–<25 kg/m^2^), and overweight (BMI ≥ 25 kg/m^2^). For children and adolescent HHCs < 20 years, we used WHO age- and gender-specific BMI z-scores tables to classify those with BMI z-score < −2 as underweight and those with z-score > 2 as overweight [19]. We conducted a principal components analysis that included housing type, number of rooms, water supply, sanitation facilities, lighting, composition of exterior walls and floor, and roof materials weighted by household size to compute a socioeconomic status (SES) score, which was then categorized into tertiles [20]. We classified HHCs as ever TB infected at baseline if they reported history of TB disease or positive TST or had a TST result ≥ 10 mm at enrollment.

In Lima, Peru, the monthly average temperature ranges between 24 and 26°C from January to April and starts to decrease in May, reaching an annual low temperature (18°C) in August; and temperatures begin to rise again in October [21]. Based on this yearly pattern, we classified a year into three seasons: winter (June through September), spring (October through December), and summer (January through May).

We used univariate and multivariate conditional logistic regression models (adjusting for matching factors) to evaluate the association between baseline vitamin D status and risk of TB disease [22]. Multivariate models included baseline covariates identified a priori as potential confounders. We used complete case analysis in the regression models. Because we had previously observed that VAD increases risk of TB disease in this cohort [13], we also adjusted for retinol levels and evaluated the interaction between VAD and VDD on incident TB risk.

In sensitivity analyses, we repeated our regressions, restricting the analysis to people who developed incident TB that was diagnosed at least 60 days after enrollment of index patient and their matched controls. We also performed a sensitivity analysis in which we included only patients with microbiologically confirmed TB and their matched controls.

Based on peer review feedback, we further examined the association between VDD and risk of incident TB diagnosed less than 60 days after index case enrollment.

Data were analyzed using SAS v9.4 (SAS Institute, Cary, NC, USA, 2013)

### Systematic review and IPD meta-analysis

Upon completing our nested case-control study analysis, we conducted a systematic review of prior prospective studies investigating the association between vitamin D status and incident TB disease. After identifying fewer than 10 studies, most of which were conducted in small cohorts and used different thresholds for categorizing vitamin D, we undertook an IPD meta-analysis to harmonize the definition of VDD and increase the power to detect differences in risk of TB by vitamin D status.

#### Search strategy and data sources

We conducted the systematic review and meta-analysis according to the Preferred Reporting Items for Systematic Reviews and Meta-Analyses (PRIMSA) guidelines (S2 Text, S3 Text) [23,24]. All studies included in the IPD meta-analysis received relevant institutional or country-specific ethics approval, and participants provided written or oral informed consent.

We initially searched the PubMed database via the NCBI Entrez system (https://www.ncbi.nlm.nih.gov/pubmed) and Embase (https://www.embase.com) for all available studies up to December 31, 2017, on the association between vitamin D and incident TB disease. Box 1 provides details of our search strategy.

**Box 1. Search strategy for studies on the association between vitamin D and incident TB disease**

**PubMed**

Mesh Terms:

1 “Vitamin D” OR “Vitamin D deficiency” AND
2 Tuberculosis

OR

Text Terms:

3 “Vitamin D” OR “Vitamin D deficiency” OR “hypovitaminosis D” OR “25-hydroxyvitamin D” OR “1,25-dihydroxyvitamin D” OR “Vitamin D2” OR “Vitamin D3” OR “Ergocalciferol” OR “Cholecalciferol” AND
4 Tuberculosis

**Embase**

Text Terms:

1 “Vitamin D” OR “Vitamin D deficiency” OR “hypovitaminosis D” OR “25-hydroxyvitamin D” OR “1,25-dihydroxyvitamin D” OR “Vitamin D2” OR “Vitamin D3” OR “Ergocalciferol” OR “Cholecalciferol” AND
2 Tuberculosis

We updated the search during peer review process to identify additional eligible studies published between January 1, 2018, and June 8, 2019.

#### Study eligibility and inclusion criteria

We placed no restrictions on language. We included all prospective longitudinal studies of human participants at risk for TB if the study measured vitamin D levels in baseline blood samples obtained prior to a diagnosis of TB disease. Studies were included if they confirmed TB diagnosis by microbiological criteria or specified their clinical criteria for ascertaining TB disease and if they collected data on age and gender. We considered any clinical criteria for TB disease based on physician assessment, imaging studies, or national guidelines. We did not place restrictions on laboratory method used to assess vitamin D levels and did not require studies to control for possible confounders. We also considered reports from conference abstracts. We excluded the following: case reports, animal or in vitro studies, case-control or cross-sectional studies that measured vitamin D levels among individuals already diagnosed with TB disease, studies that did not report vitamin D levels, studies of other diseases or non-TB-related outcomes, studies of vitamin D and TB treatment outcomes or TB infection or TB immune reconstitution inflammatory syndrome (IRIS), reviews, meta-analyses, letters, editorials, and protocols.

#### Data collection and data items

For each eligible study, two reviewers (OA and SC) independently performed full text reviews and extracted the following information: first author’s last name, year of publication, study design and study aim, country of study, calendar years of study, length of follow-up, number of incident TB cases, total number of subjects analyzed, criteria for diagnosing TB disease, laboratory method of vitamin D assay, method of categorizing vitamin D, assessment of HIV status, and covariates in multivariate analyses. Discrepancies were resolved by consensus.

We contacted all authors of the eligible studies identified from the systematic review and requested IPD. Lead investigators from all the studies agreed to provide data for analysis. Data were de-identified prior to transfer via email. We requested all available data on possible confounders of the association between vitamin D and TB disease, including age, gender, HIV status, weight, height, BMI, IPT, baseline TST result, TB disease history, and comorbid diseases. We also requested baseline vitamin D levels, incident TB disease status during study follow-up, time from enrollment to TB diagnosis, and index case smear status, if applicable. Data were reviewed for consistency with published data, and authors were contacted for clarifications or missing information as needed.

We assessed study quality and risk of bias using the Newcastle-Ottawa Scale (NOS) for assessing quality of nonrandomized studies in meta-analyses; the NOS evaluates observational studies on participant selection, comparability of study groups, and ascertainment of exposure or outcome [25]. Based on the 9-point scoring scale, we classified study quality as good (≥7 points), fair (5–6 points), and poor (<5 points).

#### Statistical analysis

We conducted a one-step IPD meta-analysis combining data from the eligible studies and from the Peru-based study reported above. We used the unified criteria described above to categorize BMI and to define VDD as serum 25–(OH)D less than 50 nmol/L and insufficiency as 50–75 nmol/L. We further defined severe VDD as 25–(OH)D less than 25 nmol/L. To account for geographic variability, we considered datasets from each single-country study and from each country within a multicountry study as independent data sources. We used generalized linear mixed univariate and multivariate models to evaluate the association between baseline vitamin D status and risk of incident TB, including an indicator for each independent dataset as a random effect to account for within-study correlation. Multivariate models were adjusted for age, gender, BMI, and HIV status. Given sparse data on other variables, we did not adjust for any other potential confounders. We also separately evaluated the association between severe VDD, compared to sufficient levels, and TB disease risk. To determine whether the effect of vitamin D on incident TB differed by HIV status, we conducted a stratified analysis. In a sensitivity analysis, we restricted the main analysis to incident TB cases diagnosed at least 60 days after enrollment. We calculated the R_one-step_^2^ statistic to assess heterogeneity [26].

The IPD meta-analysis was conducted using the R package "lme4” [27]. Datasets for the Lima cohort study and IPD meta-analysis provided as supporting information (S1 Data, S2 Data).

## Results

### Lima cohort study

**Fig 1:**
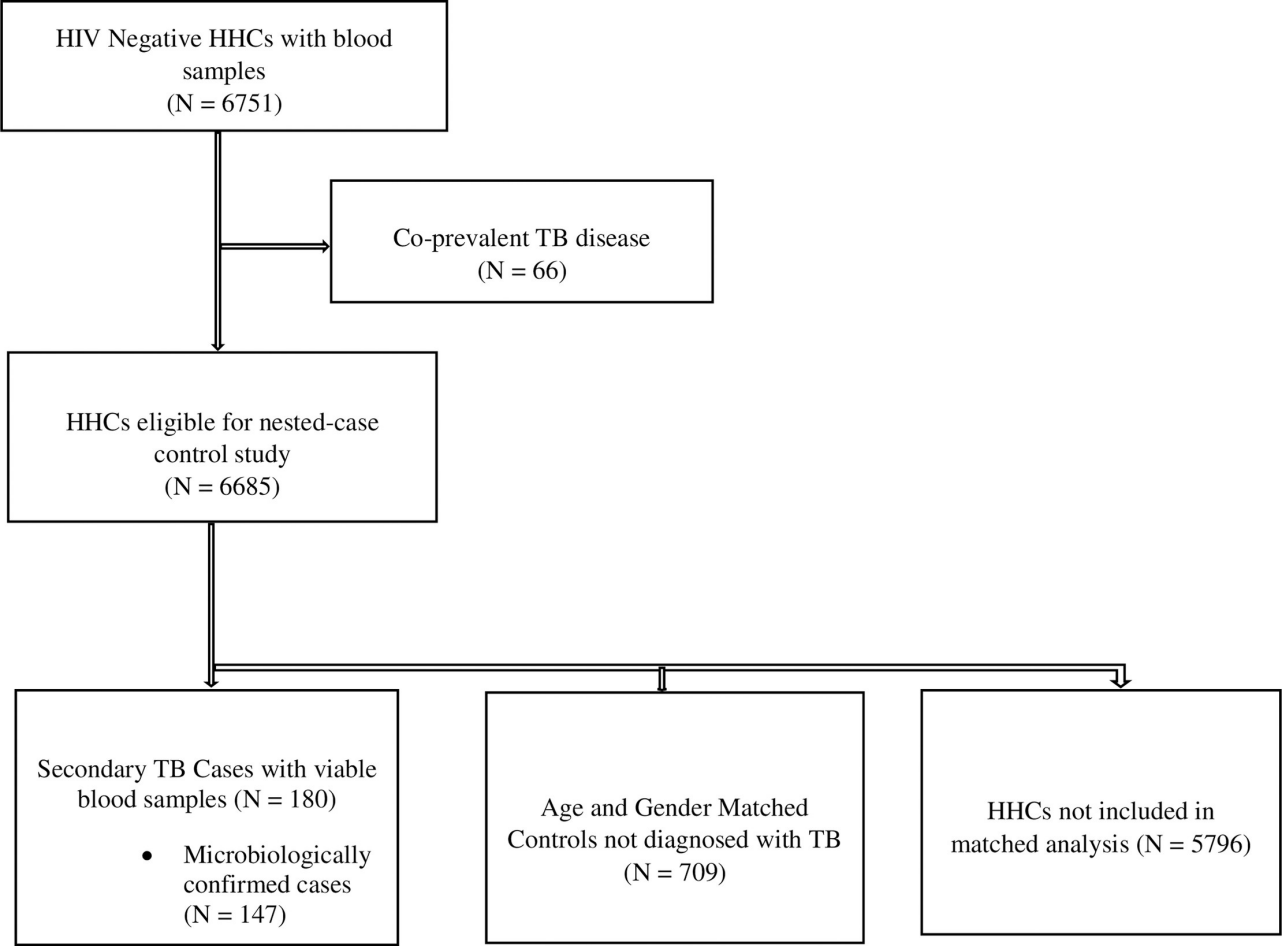
Flow diagram for selection of cases and controls in Lima cohort. HHC, household contact; TB, tuberculosis.

Among 6,751 HIV-negative HHCs with baseline blood samples, 258 developed TB disease, 66 within 15 days of enrollment and 192 thereafter. Among these 192 secondary TB cases, 152 (79.1%) were microbiologically confirmed, and viable blood samples were available for analysis for 180 (93.8%) at the end of follow-up (Fig 1). Monthly incident of TB cases is shown in S1 Fig. Among the 180 TB cases analyzed, 147 (81.7%%) were microbiologically confirmed. Table 1 lists baseline characteristics of the incident cases and their matched controls. The median levels of 25–(OH)D at baseline were similar among cases (53.9 nmol/L; interquartile range [IQR] 42.7–64.0 nmol/L) and controls (54.7 nmol/L; IQR 44.5–67.1 nmol/L; *p* = 0.32) (Table 2). Median serum 25–(OH)D levels during spring/summer (57.2 nmol/L; IQR 46.1–69.0 nmol/L) were higher than during winter (49.6 nmol/L; IQR 40.5–59.9 nmol/L; *p <* 0.001) (S2 Fig).

**Table 1:** Baseline characteristics of participants in Lima cohort study.

Characteristic	Cases, *n* (%)	*N*^a^ (Cases)	Controls, *n* (%)	*N*^a^ (Controls)	Nonmatched Household Contacts, *n* (%)	*N*^a^ (Household Contacts)
Age categories in years		180		709		5,796
0–14	15 (8.3)		60 (8.5)		600 (10.4)	
15–24	74 (41.1)		298 (42.0)		1,380 (23.8)	
≥25	91 (50.6)		351 (49.5)		3,816 (65.8)	
Male	94 (52.2)		366 (51.6)		2,413 (41.6)	
BMI categories^b^		179		706		5,745
Underweight	8 (4.5)		6 (0.9)		64 (1.1)	
Overweight	45 (25.1)		299 (42.4)		2,881 (50.2)	
Normal	126 (70.4)		401 (56.8)		2,800 (48.7)	
SES^c^		171		698		5,607
Lowest tertile	77 (45.0)		228 (32.7)		1,847 (32.9)	
Middle tertile	66 (38.6)		326 (46.7)		2,565 (45.8)	
Highest tertile	28 (16.4)		144 (20.6)		1,195 (21.3)	
Heavy alcohol use^d^	14 (8.1)	174	64 (9.3)	692	444 (7.9)	5,632
Current smoking	13 (7.4)	176	78 (11.2)	699	488 (8.5)	5,711
Self-reported diabetes	6 (3.4)	179	11 (1.6)	701	135 (2.4)	5,739
Comorbid disease^e^	37 (20.6)	180	175 (24.7)	709	1,380 (23.8)	5,795
Isoniazid preventive therapy	7 (3.9)	180	108 (15.2)	709	713 (12.3)	5,790
BCG scar	159 (88.3)	180	628 (88.6)	709	5,133 (88.6)	5,795
History of TB	34 (18.9)		55 (7.8)	708	545 (9.4)	5,782
Baseline TST positive (≥10 mm)	85 (71.4)	119	255 (39.4)	647	2,498 (46.6)	5,362
Ever TB infected at baseline	145 (82.4)	176	281 (41.1)	683	2,768 (49.0)	5,649
Mean days to TB diagnosis (± SD)	118.5 (±114.5)	180	NA		NA	
Median days to TB diagnosis (IQR)	66.0 (20.5–198.0)	180	NA		NA	
Season of blood sample collection		180		709		5,796
Spring/summer	136 (75.6)		483 (68.1)		3,821 (65.9)	
Winter	44 (24.4)		226 (31.9)		1975 (34.1)	
**Index Patient Characteristics**						
Smear positive	156 (86.7)	180	486 (68.7)	707	4,150 (71.6)	5,793
Cavitary disease	54 (30.2)	179	175 (25.1)	697	1,424 (24.9)	5,722

^a^ Total number of subjects with data for corresponding variable.

^b^ We classified adults ≥ 20 years old as underweight (BMI < 18.5 kg/m^2^), normal weight (BMI 18.5–<25 kg/m^2^), and overweight (BMI ≥ 25 kg/m^2^). For children and adolescents < 20 years old, we used WHO age- and gender-specific BMI z-scores tables to classify those with BMI z-score < –2 as underweight and those with z-score > 2 as overweight.

^c^ We conducted a principal components analysis that included housing type, number of rooms, water supply, sanitation facilities, lighting, composition of exterior walls and floor, and roof materials weighted by household size to compute an SES score, which was then categorized into tertiles [20].

^d^ Self-reported consumption of ≥40 g or ≥3 alcoholic drinks daily.

^e^ Heart disease, high blood pressure, asthma, kidney disease, use of steroids or chemotherapy or immunosuppressant, any other self-reported chronic illness.

Abbreviations: BCG, Bacillus Calmette–Guérin; BMI, body mass index; IQR, interquartile range; SES, socioeconomic status; TB, tuberculosis; TST, tuberculin skin test; WHO, World Health Organization

**Table 2:** Baseline levels of vitamin D among cases and controls in Lima cohort study.

Vitamin D levels	Cases (*N =* 180) Median (IQR) or *n* (%)	Control (*N =* 709) Median (IQR) or *n* (%)	*p*-Value^a^
25–OH Vitamin D (nmol/L)	53.9 (42.7–64.0)	54.7 (44.5–67.1)	0.32
Vitamin D deficient (<50 nmol/L)	76 (42.2)	259 (36.5)	0.13
Vitamin D insufficient (50–75 nmol/L)	84 (46.7)	348 (49.1)	0.45
Vitamin D sufficient (>75 nmol/L)	20 (11.1)	102 (14.4)	1.00

^a^ Univariate p values adjusted for matching factors (age and sex).

Abbreviation: IQR, interquartile range

In the univariate analysis, the differences in the risk of incident TB disease among HHCs with baseline VDD and baseline VDI compared to those with sufficient levels were not statistically significant (odds ratio [OR] VDD 1.54; 95% CI 0.88–2.71; *p* = 0.13 and OR VDI 1.23; 95% CI 0.72–2.08; *p* = 0.45) (Table 3). After we adjusted for BMI categories, SES, heavy alcohol consumption, tobacco use, IPT, TB infection status, comorbid disease, self-reported DM, index patient smear status, and season of sample collection, compared to those with sufficient vitamin D levels, the ORs for HHCs with baseline VDD and VDI were 1.63 (95% CI 0.75–3.52; *p* = 0.22) and 1.12 (95% CI 0.57–2.23; *p* = 0.75), respectively (Table 3). When we further adjusted for VAD, the ORs for HHCs with VDD and VDI were 1.59 (95% CI 0.71–3.55; *p* = 0.26) and 1.12 (95% CI 0.55–2.27; *p* = 0.75), respectively. A test for interaction between VAD and VDD was not statistically significant (*p* for interaction = 0.07) (S1 Table).

**Table 3:** Association between vitamin D status and risk of TB disease among household contacts of TB patients in Lima cohort study.

Vitamin D status	Cases/Controls	Univariate OR (95% CI) *N =* 889	*p*-Value	Multivariate OR^a^ (95% CI) *N =* 822	*p*-Value
Vitamin D deficient (<50 nmol/L)	76/259	1.54 (0.88–2.71)	0.13	1.63 (0.75–3.52)	0.22
Vitamin D insufficient (50–75 nmol/L)	84/348	1.23 (0.72–2.08)	0.45	1.12 (0.57–2.23)	0.74
Vitamin D sufficient (>75 nmol/L)	20/102	1.00		1.00	

^a^ Adjusted for matching factors (age and sex), BMI categories, socioeconomic status, heavy alcohol consumption, tobacco use, isoniazid preventive therapy, ever TB infected, comorbid disease, self-reported DM, index patient smear status, and season of sample collection.

Abbreviations: BMI, body mass index; DM, type 2 diabetes mellitus; OR, odds ratio; TB, tuberculosis

Our conclusions did not differ from results of the main analysis when we restricted our analyses to cases (and matched controls) diagnosed at least 60 days after index patient enrollment or to microbiologically confirmed TB cases (Table 4). We did not find a statistically significant association between VDD and risk of TB diagnosed less than 60 days after index case enrollment (OR 0.98; 95% CI 0.20–4.72; *p* = 0.98).

**Table 4:** Vitamin D status and risk of TB disease stratified by date of TB diagnosis and microbiologically confirmed TB in Lima cohort study.

Timing of TB diagnosis or TB type	Cases/Controls	Multivariate OR^a^ (95% CI)	*p*-Value
**TB diagnosed < 60 days**			
Vitamin D deficient (<50 nmol/L)	50/172	0.98 (0.20–4.72)	0.98
Vitamin D insufficient (50–75 nmol/L)	34/137	1.12 (0.28–4.45)	0.88
Vitamin D sufficient (>75 nmol/L)	5/37	1.00	
**TB diagnosed ≥ 60 days**			
Vitamin D deficient (<50 nmol/L)	26/87	1.89 (0.70–5.11)	0.21
Vitamin D insufficient (50–75 nmol/L)	50/211	1.21 (0.52–2.80)	0.65
Vitamin D sufficient (>75 nmol/L)	15/65	1.00	
**Microbiologically confirmed TB disease**			
Vitamin D deficient (<50 nmol/L)	60/209	1.91 (0.78–4.70)	0.16
Vitamin D insufficient (50–75 nmol/L)	72/284	1.36 (0.61–3.07)	0.45
Vitamin D sufficient (>75 nmol/L)	15/84	1.00	

^a^ Adjusted for matching factors (age and sex), BMI categories, socioeconomic status, heavy alcohol consumption, tobacco use, isoniazid preventive therapy, ever TB infected, comorbid disease, self-reported DM, index patient smear status, and season of sample collection.

Abbreviations: BMI, body mass index; DM, type 2 diabetes mellitus; OR, odds ratio; TB, tuberculosis

### Systematic review and IPD meta-analysis

**Fig 2:**
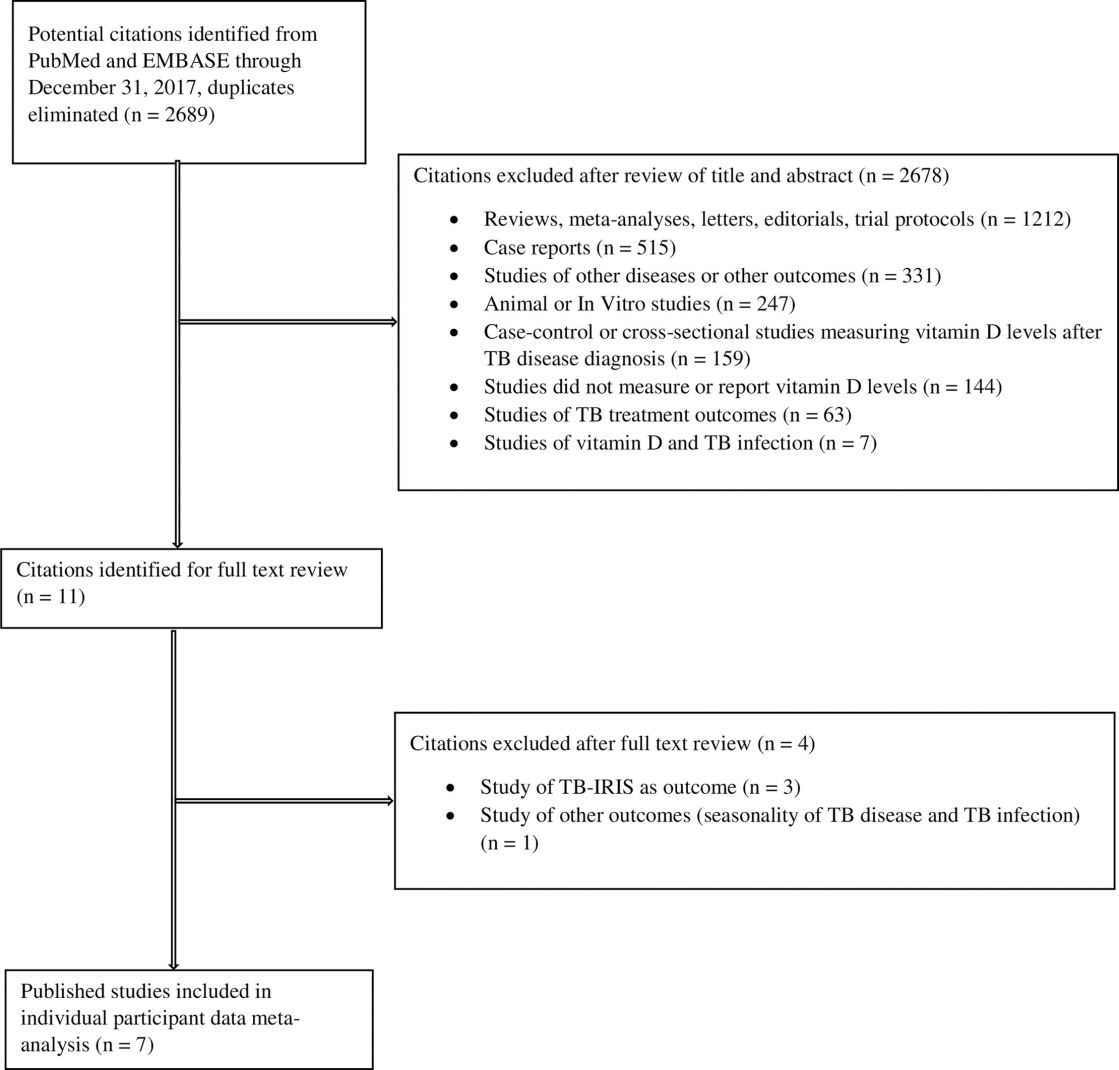
Flow diagram for selection of studies for the IPD meta-analysis. IPD, individual-participant data; IRIS, immune reconstitution inflammatory syndrome; TB, tuberculosis.

We identified 2,689 citations from the initial PubMed and Embase searches through December 31, 2017. After screening titles and abstracts, we excluded 2,678 articles because they were reviews, meta-analyses, letters, editorials or protocols (*n =* 1,212), case reports (*n =* 515), studies of other diseases or other outcomes (*n =* 331), animal or in vitro studies (*n =* 247), case-control or cross-sectional studies that assessed vitamin D status after TB disease diagnosis (*n =* 159), studies that did not measure vitamin D (*n =* 144), studies of TB treatment outcomes (*n =* 63), and studies of TB infection (*n =* 7) (Fig 2). We reviewed full texts of the remaining 11 articles [28–38] and further excluded three studies that assessed outcomes of TB-IRIS [35–37] and one study of TB infection with seasonality of TB [38]. Table 5 provides information about the seven eligible published studies [28–34] identified from the systematic review through December 31, 2017. All seven studies included in the IPD meta-analysis attained at least seven points on the NOS scale and were categorized as “good quality” studies (S2 Table).

**Table 5:** Summary of studies included in the IPD meta-analysis.

**Study [Reference]**	Country (Latitude)	Study Design and Study Population	Total Number of Participants	Number of TB Cases (%)	Median Age, Years (IQR)	Female, *N* (%)	HIV-Positive Cases, *N* (%)	Method of Measuring Vitamin D	Median Baseline 25-OH Vitamin D, nmol/L (IQR)	Length of Follow-up, Years^a^	TB Disease Definition	Adjusted Effect Estimate (95% CI) Reported From Original Study
Arnedo-Pena et al., 2015 [28]	Spain (40.4637° N, 3.7492° W)	Prospective cohort study of household and community contacts of TB cases	523	3 (0.6)	37.0 (28.0–46.0)	255 (48.8)	NA^b^	ECLIAs and CLIAs	60.0 (42.5–79.3)	Mean 1.6 (± 0.9)	Smear or culture positive	aHR for continuous vitamin D and microbiologically confirmed TB: 0.88 (0.80–0.97)
Gupta et al., 2016 [29]	South Africa (30.5595° S, 22.9375° E)	Prospective case-cohort study of HIV-positive and HIV-exposed infants (no previous known TB exposure)	366	100 (27.3)	0.7 (0.5–0.7)	196 (53.6)	193 (52.7)	Immunoassay	90.8 (75.8–109.0)	3.7	2004 South African NTP criteria for definite, probable, or possible TB^c^	aHR for vitamin D < 80 nmol/L and any TB: 1.76 (1.01–3.05)
Mave et al., 2015 [30]	India (20.5937° N, 78.9629° E)	Nested case-control study of HIV-positive breastfeeding mothers (TB exposure status not specified)	120	33 (27.5)	23.0 (21.0–25.0)	120 (100.0)	120 (100.0)	Radioimmunoassay	39.4 (24.4–47.5)	1.0	Culture confirmed OR Probable TB: (1) smear positive; and (2) histological and clinical features suggestive of TB and response to anti-TB treatment	aOR for vitamin D < 50 nmol/L and any TB: 1.57 (0.49–4.98)
Owolabi et al., 2016 [31]^d^	The Gambia (13.4432° N, 15.3101° W)	Prospective cohort study of household contacts of TB cases	139	12 (8.6)	24.0 (20.0–37.0)	72 (51.8)	0 (0.0)	ELISA	47.4 (37.4–56.4)	2.0	smear/culture positive	Adjusted linear regression estimate for continuous vitamin D and microbiologically confirmed TB: 3.65 (0.59–6.71)
Sudfeld et al., 2013 [32]	Tanzania (6.3690° S, 34.8888° E)	Prospective cohort study of HIV- positive patients initiating ART (TB exposure status not specified)	1,092	50 (4.6)	37.0 (32.0–42.8)	752 (68.9)	1,092 (100.0)	High-performance liquid chromatography	73.5 (60.3–86.8)	Median 1.7 (IQR 0.7–2.8)	smear positive or chest radiograph	aHR for vitamin D < 50 nmol and microbiologically confirmed TB: 2.89 (1.31–7.41)
Talat et al., 2010 [33]	Pakistan (30.3753° N, 69.3451° E)	Prospective cohort study of household contacts of TB cases	109	8 (7.3)	20.0 (15.0–35.0)	59 (54.1)	NA	ELISA	23.5 (13.8–43.5)	4.0	smear positive or chest radiograph	aHR for 1-log decrement in continuous vitamin D and any TB: 5.1 (1.2–21.3)
Tenforde et al., 2017 [34]	Brazil (14.2350° S, 51.9253° W), Haiti (18.9712° N, 72.2852° W), India (20.5937° N, 78.9629° E), Malawi (13.2543° S, 34.3015° E), Peru (9.1900° S, 75.0152° W), South Africa (30.5595° S, 22.9375° E), Thailand (15.8700° N, 100.9925° E), US (37.0902° N, 95.7129° W), Zimbabwe (19.0154° S, 29.1549° E)	Prospective case-cohort study of HIV-positive patients initiating ART (TB exposure status specified by history of TB disease)	306	70 (22.9)	35.0 (29.0–41.0)	141 (46.1)	306 (100.0)	Immunoassay	80.0 (57.5–97.5)	1.8	ACTG criteria for confirmed, probable, or clinical TB^e^	aHR for vitamin D < 50 nmol and any TB: 3.66 (1.16–11.51)
Lima cohort study	Peru (9.1900° S, 75.0152° W)	Nested case-control study of HIV-negative household contacts of TB cases	889	180 (20.2)	24.0 (18.0–37.0)	429 (48.3)	0 (0.0)	Immunoassay	54.5 (44.1–66.8)	1.0	Peru’s NTP criteria for TB diagnosis [15]	aOR for vitamin D < 50 nmol and any TB: 1.70 (0.84–3.46) aOR for vitamin D < 50 nmol and microbiologically confirmed TB: 1.78 (0.79–4.03)

^a^ Data as reported from original study either in total length or median/mean length.

^b^ Three incident TB cases were HIV-negative; otherwise, HIV status is not available for remaining study participants.

^c^ The South African National Tuberculosis Control Program: Practical Guidelines 2004. Available from: http://www.kznhealth.gov.za/chrp/documents/Guidelines/Guidelines%20National/Tuberculosis/SA%20TB%20Guidelines%202004.pdf

^d^ Original study reported results from case-control analysis but baseline vitamin D levels for entire cohort of household contacts provided for individual participant data meta-analysis.

^e^ Campbell TB, Smeaton LM, Kumarasamy N, Flanigan T, Klingman KL, Firnhaber C, et al. Efficacy and safety of three antiretroviral regimens for initial treatment of HIV-1: a randomized clinical trial in diverse multinational settings. PLoS Med. 2012;9(8): e1001290.

Abbreviations: ACTG, AIDS Clinical Trial Group; aHR, adjusted hazard ratio; aOR, adjusted OR; ART, antiretroviral therapy; CLIA, chemiluminescence immunoassay; ECLIA, electrochemiluminescence immunoassay; IPD, individual-patient data; IQR, interquartile range; NTP; OR, odds ratio; TB, tuberculosis

In the updated search during peer review, we identified one additional eligible study published between January 1, 2018, and June 8, 2019, that had not been included in the IPD meta-analysis [39]. Details are provided as supporting information (S3 Fig, S3 Table).

We obtained IPD from all eligible seven studies published through December 31, 2017. One study provided patient data from a multisite evaluation conducted in nine countries [34]. Six of the seven studies were prospective cohort or case-cohort studies [28, 29, 31–34], whereas one study was a nested case-control study [30]. The final combined dataset with our Lima cohort study included 3,544 participants from 13 countries: Brazil, The Gambia, Haiti, India, Malawi, Pakistan, Peru, South Africa, Spain, Tanzania, Thailand, US, and Zimbabwe. We analyzed a total of 456 TB cases. The median time to TB diagnosis from enrollment was 151.0 days (IQR 44.0–342.0 days). Table 6 lists the baseline characteristics of all patients analyzed. The majority of the participants (86.5%) were over 15 years of age. HIV status was unknown for 629 (17.7%) patients, whereas 1,711 (48.3%) were HIV positive. One study assessed serum 25–(OH)D levels using HPLC [32], whereas others used immunoassay [28–30,34] and ELISA [31,33]. The median baseline level of 25–(OH)D was 65.0 nmol/L (IQR 48.8–83.5 nmol/L). The prevalence of VDD at baseline was 26.2% and of severe VDD was 4.8%. Most of the participants with severe VDD were from studies conducted in Pakistan (35.7%) [33], Spain (25.2%) [28], and India (17.5%) [30]. Median serum 25–(OH)D levels were higher among HIV-positive participants (74.3 nmol/L; IQR 58.0–90.0 nmol/L) compared to HIV-negative individuals (56.5 nmol/L; IQR 44.6–72.5 nmol/L; *p <* 0.0001). The studies in the IPD meta-analysis did not collect similar covariates; therefore, we did not compare additional baseline variables by HIV status.

**Table 6:** Baseline demographic and clinical characteristics of participants in the IPD meta-analysis (*N =* 3,544).

Characteristic	*n* (%) or Median (IQR)
Male	1,520 (42.9)
Age categories in years	
0–14	478 (13.5)
15–24	688 (19.4)
≥25	2,378 (67.1)
HIV	
Positive	1,711 (48.3)
Negative	1,204 (34.0)
Unknown	629 (17.7)
BMI categories^a^	
Underweight	433 (12.2)
Overweight	873 (24.6)
Normal	1,858 (52.4)
Unknown	380 (10.7)
Isoniazid preventive therapy	
Yes	366 (10.3)
No	2,504 (70.7)
Unknown	674 (19.0)
Baseline tuberculin skin test	
Positive (≥10 mm)	750 (21.2)
Negative	965 (27.2)
Unknown	1,829 (51.6)
History of TB	
Yes	558 (15.7)
No	2,447 (69.1)
Unknown	539 (15.2)
Comorbid disease^b^	
Yes	1,224 (34.5)
No	1,181 (33.3)
Unknown	1,139 (32.1)
Index smear status among studies of household contacts of TB cases^c^	
Positive	1,155 (69.6)
Negative	364 (21.9)
Unknown	141 (8.5)
Antiretroviral therapy use among HIV positive^d^	
Yes	1,588 (92.8)
No	121 (7.1)
Unknown	2 (0.1)
Baseline CD4 count among HIV positive (cell/μL)^e^	167 (82–272)
25–OH vitamin D (nmol/L)	65.0 (48.8–83.5)
Vitamin D deficient (<50 nmol/L)	930 (26.2)
Vitamin D insufficient (50–75 nmol/L)	1,357 (38.3)
Vitamin D sufficient (>75 nmol/L)	1,257 (35.5)

^a^ We classified adults ≥ 20 years old as underweight (BMI < 18.5 kg/m^2^), normal weight (BMI 18.5–<25 kg/m^2^), and overweight (BMI ≥ 25 kg/m^2^). For children and adolescents < 20 years old, we used WHO age- and gender-specific BMI z-scores tables to classify those with BMI z-score < −2 as underweight and those with z-score > 2 as overweight.

^b^ Presence of any other diseases or diagnoses as defined or ascertained in original study.

^c^ *N =* 1,660.

^d^ *N =* 1,711.

^e^ *N =* 1,353.

Abbreviations: BMI, body mass index; IPD, individual-patient data; IQR, interquartile range; TB, tuberculosis; WHO, World Health Organization

In the univariate analysis, baseline VDD was associated with a 49% increased risk of progression to TB disease (OR 1.49; 95% CI 1.07–2.07; *p* = 0.02), and the OR for VDI compared to vitamin D sufficiency was 1.26 (95% CI 0.95–1.66; *p* = 0.11) [Table 7]. Both VDD and VDI remained associated with an increased risk of TB disease after we adjusted for age, gender, BMI, and HIV status (adjusted OR [aOR] for VDD: 1.48; 95% CI 1.04–2.10; *p* = 0.03; R^2^ = 0.97 and aOR for VDI: 1.33; 95% CI 1.00–1.78; *p* = 0.05; R^2^ = 0.98).

**Table 7:** Association between selected baseline characteristics and risk of incident TB disease in the IPD meta-analysis.

Characteristic	Univariate OR^a^ (95% CI) *N =* 3,544	*p*-Value	Multivariate OR^b^ (95% CI) *N =* 2,769	*p*-Value
BMI categories^c^				
Underweight	1.43 (1.02–2.03)	0.04	1.37 (0.95–1.95)	0.09
Overweight	0.41 (0.30–0.55)	<0.001	0.40 (0.30–0.56)	<0.001
Normal	1.00		1.00	
HIV positive^d^	1.44 (0.92–2.25)	0.11	1.22 (0.77–1.95)	0.40
Vitamin D deficient (<50 nmol/L)	1.49 (1.07–2.07)	0.02	1.48 (1.04–2.10)	0.03
Vitamin D insufficient (50–75 nmol/L)	1.26 (0.95–1.66)	0.11	1.33 (1.00–1.78)	0.05
Vitamin D sufficient (>75 nmol/L)	1.00		1.00	
		*p* trend = 0.02		*p* trend = 0.03

^a^ Adjusted for age and gender because of presence of age- and gender-matched case-control study in the combined dataset.

^b^ Adjusted for age, gender, BMI categories, and HIV status.

^c^ *N =* 3,164.

^d^ *N =* 2,915.

Abbreviations: BMI, body mass index; IPD, individual-patient data; OR, odds ratio; TB, tuberculosis

When we stratified by HIV status, HIV-positive individuals with VDD were twice as likely to develop TB disease compared to those with normal levels (aOR 2.18; 95% CI 1.22–3.90; *p* = 0.01; R^2^ = 0.97), whereas the aOR for TB disease among HIV-negative participants with VDD was 1.20 (95% CI 0.74–1.93; *p* = 0.46; R^2^ = 0.99) (Table 8, *p* for interaction = 0.17).

**Table 8:** Vitamin D deficiency and risk of incident TB disease stratified by HIV status in the IPD meta-analysis^a^.

	HIV Positive			HIV Negative		
Characteristic	*N*	Multivariate OR^b^ (95% CI)	*p*-Value	*N*	Multivariate OR^b^ (95% CI)	*p*-Value
Vitamin D deficient (<50 nmol/L)	146	2.18 (1.22–3.90)	0.01	419	1.20 (0.74–1.93)	0.46
Vitamin D insufficient (50–75 nmol/L)	623	1.32 (0.90–1.94)	0.16	509	1.18 (0.76–1.84)	0.46
Vitamin D sufficient (>75 nmol/L)	807	1.00		265	1.00	
			*p* trend = 0.01			*p* trend = 0.52
BMI categories						
Underweight	392	1.02 (0.68–1.54)	0.92	34	3.99 (1.79–8.87)	0.001
Overweight	231	0.41 (0.21–0.78)	0.01	449	0.41 (0.29–0.59)	<0.001
Normal	953	1.00		710	1.00	

*p* Test for interaction between HIV status and serum 25–(OH)D levels = 0.17.

^a^ Excluded datasets from Arnedo-Pena and colleagues [28] and Talat and colleagues [33] because of lack of information on HIV status.

^b^ Adjusted for age, gender, and BMI categories.

Abbreviations: BMI, body mass index; IPD, individual-patient data; OR, odds ratio; TB, tuberculosis

In the entire IPD cohort, the aOR for incident TB among those with severe VDD was 2.05 (95% CI 0.87–4.87; *p* trend for stepwise decrease in serum 25–(OH)D levels from sufficient to severe deficiency = 0.02; R^2^ = 0.91) (Table 9). Among HIV-positive individuals, the OR for severe VDD compared to sufficient vitamin D levels was 4.28 (95% CI 0.85–21.45; *p* = 0.08; R^2^ = 0.90). In contrast, among HIV-negative individuals, the OR for severe VDD was 1.55 (95% CI 0.55–4.34; *p* = 0.41; R^2^ = 0.97) (Table 9, *p* for interaction 0.17).

**Table 9:** Severe vitamin D deficiency and risk of incident TB disease stratified by HIV status in the IPD meta-analysis.

Vitamin D status	N	Multivariate OR (95% CI)	*p*-Value
**All participants (*N =* 2,769)**^**a**^			
Vitamin D < 25 nmol/L	38	2.05 (0.87–4.87)	0.10
Vitamin D 25–<50 nmol/L	527	1.45 (1.01–2.06)	0.04
Vitamin D insufficient (50–75 nmol/L)	1,132	1.33 (1.00–1.78)	0.05
Vitamin D sufficient (>75 nmol/L)	1,072	Ref	
			*p* trend = 0.02
**HIV positive (*N =* 1,576)**^**b**^			
Vitamin D < 25 nmol/L	12	4.28 (0.85–21.45)	0.08
Vitamin D 25–<50 nmol/L	134	2.06 (1.13–3.76)	0.03
Vitamin D insufficient (50–75 nmol/L)	623	1.32 (0.90–1.95)	0.16
Vitamin D sufficient (>75 nmol/L)	807	Ref	
			*p* trend = 0.01
**HIV negative (*N =* 1,193)**^**b**^			
Vitamin D < 25 nmol/L	26	1.55 (0.55–4.34)	0.41
Vitamin D 25–<50 nmol/L	393	1.17 (0.73–1.90)	0.51
Vitamin D insufficient (50–75 nmol/L)	509	1.18 (0.76–1.84)	0.46
Vitamin D sufficient (>75 nmol/L)	265	Ref	
			*p* trend = 0.46

*p* Test for interaction between HIV status and serum 25–(OH)D levels = 0.17.

^a^ Model adjusted for age, gender, BMI categories, and HIV status.

^b^ Model adjusted for age, gender, BMI, categories.

Abbreviations: BMI, body mass index; IPD, individual-patient data; OR, odds ratio; TB, tuberculosis

When we separately considered incident cases diagnosed at least 60 days after enrollment, VDI remained associated with increased risk of TB disease (aOR 1.40; 95% CI 1.02–1.92; *p* = 0.04; R^2^ = 0.97), whereas VDD was no longer associated with incident TB (aOR 1.02; 95% CI 0.67–1.57; *p* = 0.92; R^2^ = 0.95). Only 51.1% of incident TB cases in the IPD meta-analysis cohort had data on microbiologic confirmation, and we therefore did not conduct a sensitivity analysis.

In the one publication we identified that appeared after the target dates for the IPD meta-analysis, Maceda and colleagues [39] reported on a nested case-control study of 72 male prisoners in Brazil. Mean 25–(OH)D levels did not differ significantly among cases (92.5 ± 37.0 nmol/L) and controls (93.8 ± 27.5 nmol/L), and there was no association between serum 25–(OH)D < 75 nmol/L and risk of incident TB disease during 1 year of follow-up (aOR 0.59; 95% CI 0.13–2.62) (S3 Table).

## Discussion

Our IPD meta-analysis provides consistent support for a modest dose-dependent effect of vitamin D on future progression of TB disease across multiple studies conducted in diverse contexts. In the IPD, the association of low serum 25–(OH)D levels with increased TB disease risk was most pronounced among HIV-positive individuals with severe VDD.

Although some previous studies have documented lower vitamin D levels among patients with active TB compared to healthy controls [9,10,12,40], others have not confirmed the association between VDD and increased TB disease risk [11]. Furthermore, even among studies that found lower vitamin D levels in TB patients compared to healthy controls, the causal direction of this association is difficult to infer, since TB disease can lead to reduced dietary intake and micronutrient deficiencies, which resolve with successful treatment [10]. Although a number of intervention studies have found little evidence of an impact of vitamin D supplementation on TB treatment outcomes [10,41–46], a recent study demonstrated improved outcomes in a subgroup of multidrug-resistant patients who received supplementation [47]. In contrast to studies of micronutrients and TB treatment outcomes [10,41–46], relatively few studies have prospectively investigated the role of preexisting vitamin D status in the development of TB disease.

Our findings in these human studies support the role of vitamin D in TB infection and disease that has been inferred from more fundamental research that has enumerated multiple mechanisms by which VDD modulates host immune response to MTB. In macrophages, vitamin D is implicated in the activation of cathelicidin-mediated killing of ingested mycobacteria [5,48] induction of IFN-γ-mediated activity in macrophages [6], induction of reactive oxygen and nitrogen species [7], stimulation of phagolysosome fusion in infected macrophages [8], and inhibition of matrix metalloproteinases involved in the pathogenesis of cavitary pulmonary TB [49]. Recent evidence has also demonstrated vitamin D is involved in reduced dendritic cell–mediated priming of the adaptive immune response [50].

In addition to its impact on immunity, vitamin D status has also been linked to human metabolic phenotypes that may be involved in the pathogenesis of TB. In vitro studies have demonstrated various ways by which vitamin D promotes insulin sensitivity [51], and animal models have shown VDD impairs insulin secretion in pancreatic beta cells [51,52]. Numerous observational studies have also found an inverse association between vitamin D levels and incident type 2 diabetes mellitus (DM) [51]. Given DM is a well-described risk factor for TB disease [53,54], VDD may also contribute to increased TB risk through its role in modifying risk of diabetes.

Two other lines of evidence point to a possible association between vitamin D and TB. First, multiple studies have reported an association between specific polymorphisms of vitamin D receptor (VDR) and vitamin D binding protein (VDBP) and increased TB risk [10,55–57]. Although it is not clear that the functional effect of these polymorphisms recapitulates the impact of low vitamin D levels, several studies show that the impact of VDR variants is stronger in the presence of VDD [29,55,58]. Secondly, TB incidence varies with season and peaks in spring and summer months when vitamin D levels are highest. Some observers have postulated that low levels of sunshine, and hence vitamin D, in winter contribute to an increase in TB infection followed by a rise in TB disease incidence after a 6-month lag [38,59–61].

We also note previous studies have reported that low vitamin A is a strong predictor of incident TB disease [34, 62]. Vitamins A and D mediate changes at the cellular level by binding to nuclear hormone receptors, retinoic acid receptor, and VDR, respectively; and both receptors bind to retinoid X receptor (RXR) [63]. Some in vitro evidence further suggests vitamins A and D have synergistic activity in restricting MTB entry and reducing survival within macrophages [64]. Although we did not find evidence of a statistically significant interaction between vitamins A and D deficiencies on risk of TB, our study may not have been powered to detect this interaction. In a previous analysis of the Lima cohort, we found VAD conferred a 10-fold increase in TB disease risk [13], and here, we show that adjustment for vitamin A modestly attenuates the impact of vitamin D. Similarly, Tenforde and colleagues also reported that adjusting for retinol levels attenuated the effect of vitamin D on TB disease risk [34]. This raises the possibility that vitamin D levels correlate with other micronutrients implicated in the pathogenesis of TB, and these micronutrients may be potent mediators of increased TB risk.

Although we did not detect a statistically significant interaction between vitamin D and HIV status in the IPD, our findings raise the possibility that the effect of low serum 25–(OH)D on TB risk may be more pronounced among HIV-positive patients. Studies have shown that among HIV-infected individuals, VDD is associated with deleterious immune activation [65], lower CD4 counts [65,66], higher viral loads [65], and accelerated HIV disease progression [65,67]. Thus, VDD may exacerbate existing immune dysregulation in HIV infection to further increase TB risk, or low vitamin D levels may reflect severity of HIV-related immunosuppression. We also note that vitamin D status fluctuates with season, with declines in serum 25–(OH)D in the winter when UVB exposure is lower [68,69], and in vitro evidence suggests there is winter-associated increase in HIV replication [68]. Vitamin D also restricts mycobacterial growth in the presence of HIV infection [70]. A clinical trial is currently underway to evaluate the efficacy of vitamin D supplementation in preventing incident TB among adults with HIV in Tanzania [71]; the results may help clarify role of vitamin D in HIV-associated TB disease. We also plan to measure inflammatory markers in the Peru cohort to explore association between VDD and immune dysregulation.

We considered possible explanations for why we did not detect a significant association between VDD and TB risk in the Peru cohort, despite its relatively large size. First, since TB incidence is highest in summer [60,61] and HHCs were recruited when the index case was diagnosed, they are more likely to have been recruited and assessed when their vitamin D levels were highest. If levels later fell and this fall precipitated TB progression, this would not have been detected. Secondly, VDR variants are heterogeneously distributed in different populations and may modify the effect of vitamin D on TB risk. We did not measure VDR variants in Peru and are therefore unable to assess the prevalence of different VDR genotypes in this cohort. The Peru study is also limited by the relatively short (1-year) period of follow-up and the fact that it was only powered to detect a 3-fold or greater difference in TB incidence among people with VDD.

Our IPD meta-analysis also has some important limitations. Firstly, many possible confounding covariates were not measured across all studies. Therefore, we were unable to account for other important confounders such as baseline infection status, other micronutrient levels, and comorbidities, including DM, that might be associated with both VDD and TB risk. Secondly, although we only examined prospective studies of incident TB disease, the included studies were all observational, and we cannot exclude the possibility that participants had early, undiagnosed TB at baseline that lowered vitamin D levels. Although we addressed this by conducting a sensitivity analysis excluding incident TB cases diagnosed less than 60 days after enrollment, the smaller number of incident cases diagnosed after 60 days reduced the power to detect a statistically significant association. Thirdly, we also cannot exclude the possibility of publication bias if studies with nonsignificant findings on the link between vitamin D and incident TB have remained unpublished. We did not construct a funnel plot to assess publication bias, because we analyzed seven studies, and guidelines suggest tests for funnel plot asymmetry are not sufficiently powered to distinguish real asymmetry from chance with fewer than 10 studies [72]. These studies further used different methods to categorize vitamin D levels and therefore provided effect estimates that are not directly comparable on a funnel plot. During our systematic review, we attempted to address this by considering data reported from meeting abstracts, and none met our inclusion criteria. Of note, in the publication identified after the initial meta-analysis target dates, although Maceda and colleagues found no association between serum 25–(OH)D < 75 nmol/L and increased TB risk, our effect estimate for VDD in the IPD (OR 1.48) falls within the confidence intervals of this small cohort study [39].

We also note that the different 25–(OH)D assays employed in the meta-analysis studies vary in their sensitivity and precision. However, it is unlikely such variability introduced a bias in one direction, since within any given study, 25–(OH)D levels were analyzed using the same assay in individuals that progressed to TB and nonprogressors. The effect of any imprecision would be more likely to increase the noise:signal ratio, which would bias results of the analysis toward the null.

Despite the aforementioned limitations, we present findings from one of the largest cohorts to date evaluating the role of vitamin D on incident TB disease with comprehensive adjustment for possible confounders. The concurrent IPD meta-analysis further increased the sample size, providing the statistical power to detect more modest associations and enabling the evaluation of this relationship across different locations and by HIV status.

In conclusion, in our meta-analysis of prospective studies, we found low serum 25–(OH)D levels were associated with increased risk of future progression to TB disease in a dose-dependent manner. Randomized control trials are needed to determine whether vitamin D supplementation among individuals at high risk can mitigate the risk of developing TB disease.

## Supporting information

S1 Data Dataset for Lima cohort study. (XLSX)

S2 Data Dataset for individual participant data meta-analysis. (CSV)

S1 Text STROBE checklist. (DOC)

S2 Text PRIMSA checklist. PRISMA, Preferred Reporting Items for Systematic Reviews and Meta-Analyses.
(DOC)

S3 Text IPD checklist. IPD, individual-patient data.
(DOCX)

S1 Fig Monthly incident of TB cases in Lima cohort study. TB, tuberculosis.
(TIF)

S2 Fig Monthly variation of baseline serum 25–(OH)D levels in the Lima cohort study. (TIF)

S3 Fig Flow diagram for updated search for IPD meta-analysis. **Diagram describes process for identifying eligible studies published between January 1, 2018, and June 8, 2019, that were not included in the IPD meta-analysis.** IPD, individual-patient data.
(TIF)

S1 Table Interaction between vitamin A and vitamin D deficiencies on risk of TB disease. TB, tuberculosis.
(DOCX)

S2 Table Risk of bias and study quality assessment using the NOS. NOS, Newcastle-Ottawa Scale.
(DOCX)

S3 Table Details of study identified from updated search and not included in the individual participant data meta-analysis. (DOCX)

## Abbreviations

ACTG: AIDS Clinical Trial Group
aHR: adjusted hazard ratio
aOR: adjusted odds ratio
BCG: Bacillus Calmette–Guérin
BMI: body mass index
CLIA: chemiluminescence immunoassay
DM: type 2 diabetes mellitus
ECLIA: electrochemiluminescence immunoassay
HCC: household contact
HPLC: high-performance liquid chromatography
IFN-γ: interferon gamma
IPD: individual-participant data
IPT: isoniazid preventive therapy
IQR: interquartile range
IRIS: immune reconstitution inflammatory syndrome
MTB: *Mycobacterium tuberculosis*
NOS: Newcastle-Ottawa Scale
OR: odds ratio
PRISMA: Preferred Reporting Items for Systematic Reviews and Meta-Analyses
RXR: retinoid X receptor
SES: socioeconomic status
TB: tuberculosis
TST: tuberculin skin test
VAD: vitamin A deficiency
VDBP: vitamin D binding protein
VDD: vitamin D deficiency
VDI: vitamin D insufficiency
VDR: vitamin D receptor
WHO: World Health Organization
