# Supplementary material for: Vitamin D status and risk of incident tuberculosis disease: A nested case-control study, systematic review, and individual-participant data meta-analysis
Source: PLoS Med. 2019 Sep 11;16(9):e1002907. doi: 10.1371/journal.pmed.1002907 (PMC6738590; doi:10.1371/journal.pmed.1002907)
Supplement: S1 Text — (DOC) [file pmed.1002907.s003.doc]

STROBE Statement—Checklist of items that should be included in reports of ***case-control studies***

|  | Item No | Recommendation |
| --- | --- | --- |
| **Title and abstract** | 1 | (*a*) Indicate the study’s design with a commonly used term in the title or the abstract  **Title and Abstract, paragraph 2** |
|  |  | (*b*) Provide in the abstract an informative and balanced summary of what was done and what was found  **Abstract, paragraphs 2-3** |
| Introduction | | |
| Background/rationale | 2 | Explain the scientific background and rationale for the investigation being reported  **Introduction, Paragraphs 1-2** |
| Objectives | 3 | State specific objectives, including any prespecified hypotheses  **Introduction, Paragraph 3** |
| Methods | | |
| Study design | 4 | Present key elements of study design early in the paper  **Methods Lima Cohort Study, Study Setting and Population, Paragraphs 1–3** |
| Setting | 5 | Describe the setting, locations, and relevant dates, including periods of recruitment, exposure, follow-up, and data collection  **Methods Lima Cohort Study, Study Setting and Population, Paragraphs 1–3** |
| Participants | 6 | (*a*) Give the eligibility criteria, and the sources and methods of case ascertainment and control selection. Give the rationale for the choice of cases and controls  **Methods Lima Cohort Study, Study Setting and Population, Paragraphs 1–3** |
|  |  | (*b*) For matched studies, give matching criteria and the number of controls per case  **Methods Lima Cohort Study, Study Setting and Population, Paragraph 3** |
| Variables | 7 | Clearly define all outcomes, exposures, predictors, potential confounders, and effect modifiers. Give diagnostic criteria, if applicable  **Methods Lima Cohort Study, Statistical Analysis, Paragraphs 1 – 5** |
| Data sources/ measurement | 8* | For each variable of interest, give sources of data and details of methods of assessment (measurement). Describe comparability of assessment methods if there is more than one group  **Methods Lima Cohort Study, Statistical Analysis, Paragraphs 1 – 2** |
| Bias | 9 | Describe any efforts to address potential sources of bias  **Methods Lima Cohort Study, Statistical Analysis, Paragraph 4** |
| Study size | 10 | Explain how the study size was arrived at  **Methods Lima Cohort Study, Study Setting and Population, Paragraph 3** |
| Quantitative variables | 11 | Explain how quantitative variables were handled in the analyses. If applicable, describe which groupings were chosen and why  **Methods Lima Cohort Study, Statistical Analysis, Paragraphs 1 – 2** |
| Statistical methods | 12 | (*a*) Describe all statistical methods, including those used to control for confounding  **Methods Lima Cohort Study, Statistical Analysis, Paragraph 3** |
|  |  | (*b*) Describe any methods used to examine subgroups and interactions  **Methods Lima Cohort Study, Statistical Analysis, Paragraph 3-5** |
|  |  | (*c*) Explain how missing data were addressed  **Methods Lima Cohort Study, Statistical Analysis, Paragraph 3** |
|  |  | (*d*) If applicable, explain how matching of cases and controls was addressed  **Methods Lima Cohort Study, Study Setting and Population, Paragraph 3** |
|  |  | (*e*) Describe any sensitivity analyses  **Methods Lima Cohort Study, Statistical Analysis, Paragraph 4-5** |
| Results | | |
| Participants | 13* | (a) Report numbers of individuals at each stage of study—eg numbers potentially eligible, examined for eligibility, confirmed eligible, included in the study, completing follow-up, and analysed  **Results, Lima cohort study, Paragraph 1** |
|  |  | (b) Give reasons for non-participation at each stage  **Results, Lima cohort study, Paragraph 1** |
|  |  | (c) Consider use of a flow diagram  **Figure 1** |
| Descriptive data | 14* | (a) Give characteristics of study participants (eg demographic, clinical, social) and information on exposures and potential confounders  **Results, Lima cohort study, Paragraph 1 and Table 1** |
|  |  | (b) Indicate number of participants with missing data for each variable of interest  **Table 1** |
| Outcome data | 15* | Report numbers in each exposure category, or summary measures of exposure  **Tables 1 and 2** |
| Main results | 16 | (*a*) Give unadjusted estimates and, if applicable, confounder-adjusted estimates and their precision (eg, 95% confidence interval). Make clear which confounders were adjusted for and why they were included  **Results, Lima cohort study, Paragraph 2 and Table 3** |
|  |  | (*b*) Report category boundaries when continuous variables were categorized  **Methods Lima Cohort Study, Statistical Analysis, Paragraph 1-2** |
|  |  | (*c*) If relevant, consider translating estimates of relative risk into absolute risk for a meaningful time period  **NA** |

| Other analyses | 17 | Report other analyses done—eg analyses of subgroups and interactions, and sensitivity analyses  **Results, Lima cohort study, Paragraph 3 and Table 4** |
| --- | --- | --- |
| Discussion | | |
| Key results | 18 | Summarise key results with reference to study objectives  **Discussion, Paragraphs 1, 6, 8** |
| Limitations | 19 | Discuss limitations of the study, taking into account sources of potential bias or imprecision. Discuss both direction and magnitude of any potential bias  **Discussion, Paragraph 8** |
| Interpretation | 20 | Give a cautious overall interpretation of results considering objectives, limitations, multiplicity of analyses, results from similar studies, and other relevant evidence  **Discussion, Paragraph 2-5** |
| Generalisability | 21 | Discuss the generalisability (external validity) of the study results  **Discussion, Paragraph 11-12** |
| Other information | | |
| Funding | 22 | Give the source of funding and the role of the funders for the present study and, if applicable, for the original study on which the present article is based  **Additional Information** |

*Give information separately for cases and controls.

**Note:** An Explanation and Elaboration article discusses each checklist item and gives methodological background and published examples of transparent reporting. The STROBE checklist is best used in conjunction with this article (freely available on the Web sites of PLoS Medicine at http://www.plosmedicine.org/, Annals of Internal Medicine at http://www.annals.org/, and Epidemiology at http://www.epidem.com/). Information on the STROBE Initiative is available at http://www.strobe-statement.org.
