# Supplementary material for: Vitamin D status and risk of incident tuberculosis disease: A nested case-control study, systematic review, and individual-participant data meta-analysis
Source: PLoS Med. 2019 Sep 11;16(9):e1002907. doi: 10.1371/journal.pmed.1002907 (PMC6738590; doi:10.1371/journal.pmed.1002907)
Supplement: S3 Text — IPD, individual-patient data. (DOCX) [file pmed.1002907.s005.docx]

## **Checklist for reporting of individual participant data meta-analysis**

| **Item** | **Description** | **Response** |
| --- | --- | --- |
| 1 | Whether there was a protocol for the individual participant data project, and where it can be found | NA |
| 2 | 1. Whether ethics approval was necessary and (if appropriate) granted | Methods, Systematic Review and IPD Meta-analysis, Para 4 |
| 3 | 1. Why the individual participant data approach was initiated | Intro, para 1-2  Methods, Systematic Review and IPD Meta-analysis, Para 1 |
| 4 | 1. The process used to identify relevant studies for the meta-analysis | Methods, Systematic Review and IPD Meta-analysis, para 3, 4 |
| 5 | 1. How authors of relevant studies were approached for individual participant data | Methods, Systematic Review and IPD Meta-analysis, para 6 |
| 6 | How many authors (or collaborating groups) were approached for individual participant data, and the proportion that provided such data | Methods, Systematic Review and IPD Meta-analysis, para 6 |
| 7 | 1. The number of authors who did not provide individual participant data, the reasons why, and the number of patients (and events) in the respective study | Not applicable; all authors provided participant data |
| 8 | 1. Whether those authors who provided individual participant data gave all their data or only a proportion; if the latter, then describe what information was omitted and why | Methods, Systematic Review and IPD Meta-analysis, para 6 |
| 9 | 1. Whether there were any qualitative or quantitative differences between those studies providing individual participant data and those studies not providing individual participant data (if appropriate) | Not applicable |
| 10 | 1. The number of patients within each of the original studies and, if appropriate, the number of events | Table 5 |
| 11 | 1. Details of any missing individual level data within the available individual participant data for each study, and how this was handled within the meta-analyses performed | Table 6  Methods, Systematic Review and IPD Meta-analysis, para 8 |
| 12 | Details and reasons for including (or excluding) patients who were originally excluded (or included) by the source study investigators | Not applicable |
| 13 | Whether a one step or a two step individual participant data meta-analysis was performed, and the statistical details thereof, including how clustering of patients within studies was accounted for | Methods, Systematic Review and IPD Meta-analysis, para 8 |
| 14 | How many patients from each study were used in each meta-analysis performed | Table 5 |
| 15 | Whether the assumptions of the statistical models were validated (for example, proportional hazards) within each study | Not applicable |
| 16 | Whether the individual participant data results for each study were comparable with the published results, and, if not, why not (for example, individual participant data contained updated or modified information) | Not applicable |
| 17 | How individual participant data and non-individual participant data studies were analysed together (if appropriate). | Not applicable |
| 18 | The robustness of the meta-analysis results following the inclusion or exclusion of non-individual participant data studies (if appropriate) | Not applicable |

Riley RD, Lambert PC, Abo-Zaid G. Meta-analysis of individual participant data: rationale, conduct, and reporting .BMJ 2010;340:c221
