# Supplementary figures and images for: Vitamin D status and risk of incident tuberculosis disease: A nested case-control study, systematic review, and individual-participant data meta-analysis
Source: PLoS Med. 2019 Sep 11;16(9):e1002907. doi: 10.1371/journal.pmed.1002907 (PMC6738590; doi:10.1371/journal.pmed.1002907)

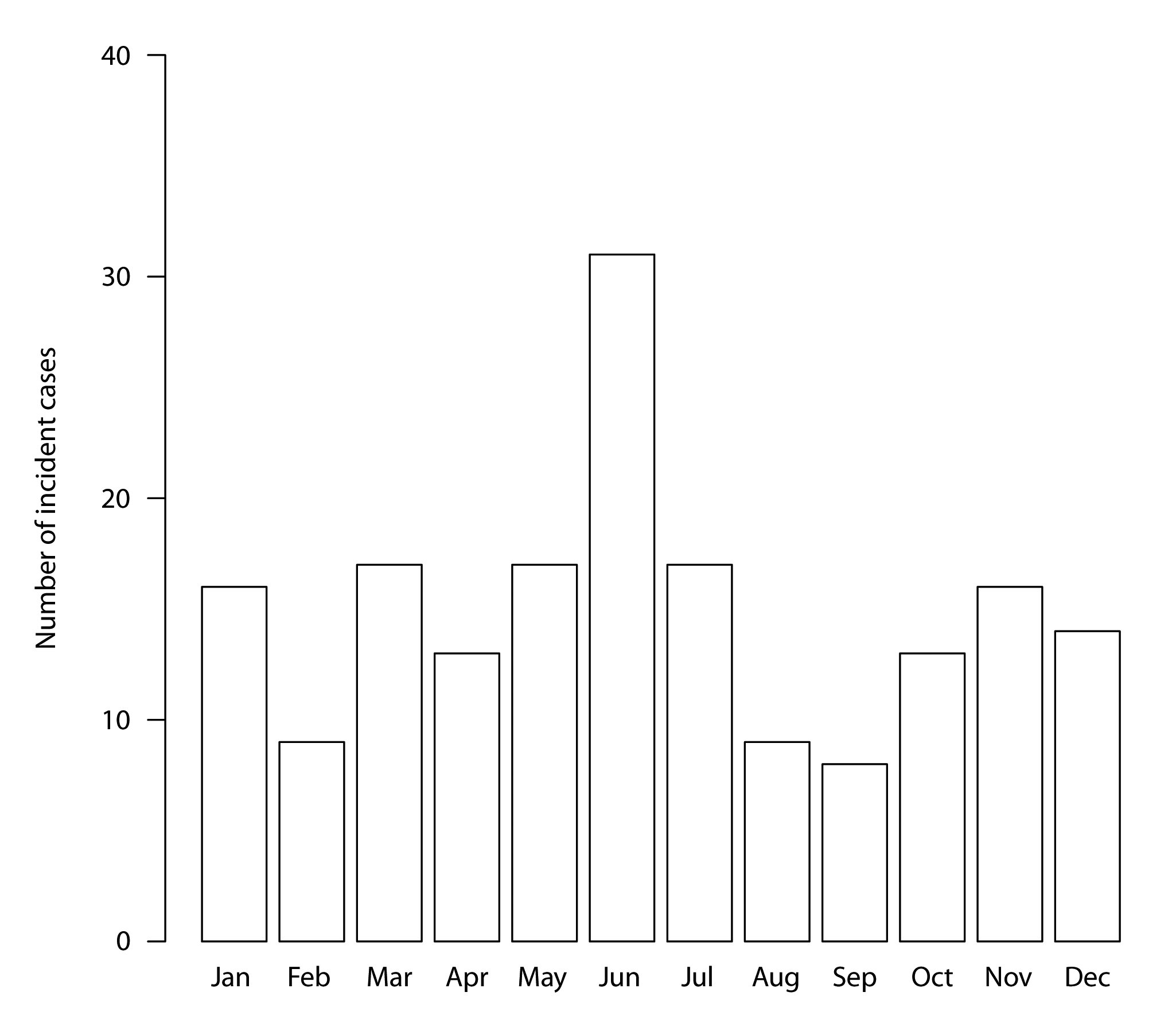

Supplement: S1 Fig — TB, tuberculosis. (TIF) [file pmed.1002907.s006.tif]

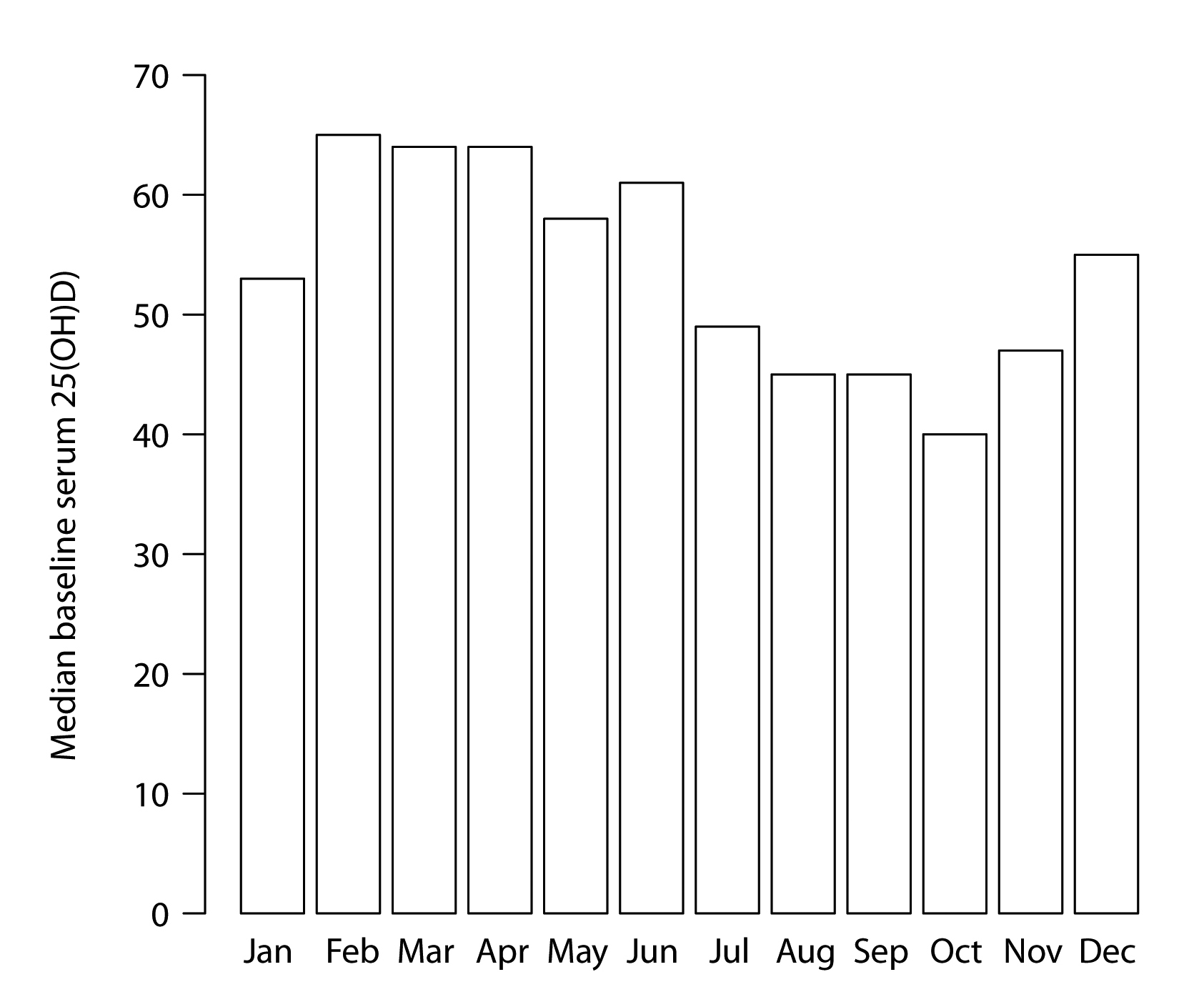

Supplement: S2 Fig — (TIF) [file pmed.1002907.s007.tif]

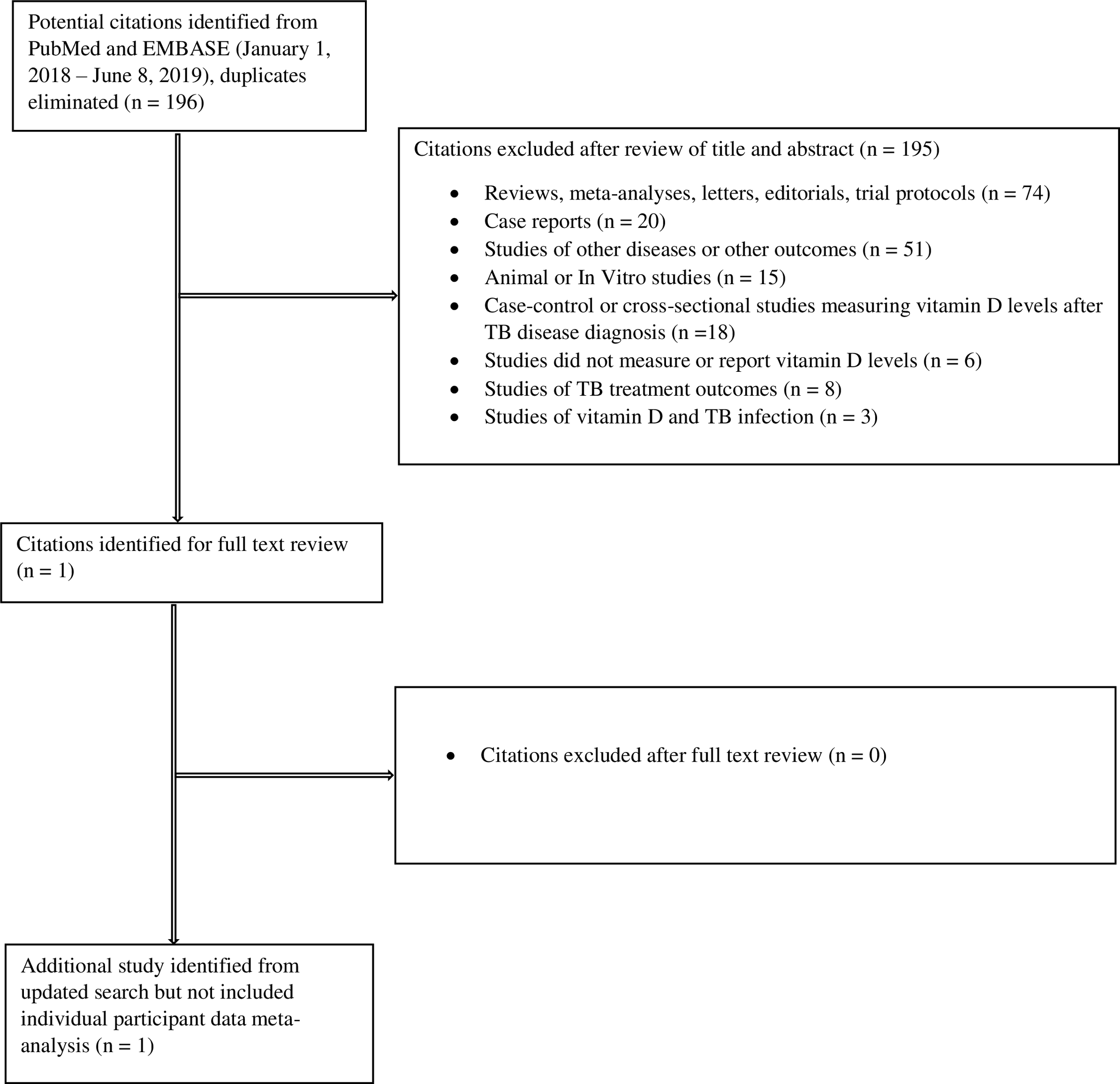

Supplement: S3 Fig — Diagram describes process for identifying eligible studies published between January 1, 2018, and June 8, 2019, that were not included in the IPD meta-analysis. IPD, individual-patient data. (TIF) [file pmed.1002907.s008.tif]
