# Supplementary material for: Vitamin D status and risk of incident tuberculosis disease: A nested case-control study, systematic review, and individual-participant data meta-analysis
Source: PLoS Med. 2019 Sep 11;16(9):e1002907. doi: 10.1371/journal.pmed.1002907 (PMC6738590; doi:10.1371/journal.pmed.1002907)
Supplement: S1 Table — TB, tuberculosis. (DOCX) [file pmed.1002907.s009.docx]

**S1 Table****. Interaction between vitamin A and vitamin D deficiencies on risk of TB disease.**

| **Vitamin D deficiency** | **Vitamin A deficiency** | **Cases/Controls** | **Multivariate OR (95% CI)^a^** | | **p value** |
| --- | --- | --- | --- | --- | --- |
| + | + | 12/9 | OR_vitamin D deficiency among household contacts with vitamin A deficiency_ | 0.18 (0.02 – 1.70) | 0.14 |
| + | - | 64/250 | OR_vitamin D deficiency among household contacts without vitamin A deficiency_ | 1.60 (0.93 – 2.75) | 0.09 |
| - | - | 92/443 |  | 1.00 |  |

p value for interaction = 0.07
^a^ Adjusted for matching factors (age and sex), body mass index (BMI) categories, socioeconomic status, heavy alcohol consumption, tobacco use, isoniazid preventive therapy, ever TB infected, comorbid disease, self-reported DM, index patient smear status, and season of sample collection.
