# Supplementary material for: Vitamin D status and risk of incident tuberculosis disease: A nested case-control study, systematic review, and individual-participant data meta-analysis
Source: PLoS Med. 2019 Sep 11;16(9):e1002907. doi: 10.1371/journal.pmed.1002907 (PMC6738590; doi:10.1371/journal.pmed.1002907)
Supplement: S2 Table — NOS, Newcastle-Ottawa Scale. (DOCX) [file pmed.1002907.s010.docx]

**S2 Table. Risk of Bias and Study Quality Assessment using the Newcastle-Ottawa Scale (NOS)^a^**

|  | **Study (reference)** | **Selection (Maximum 4 stars)** | **Comparability (Maximum 2 stars)** | **Exposure/Outcome Maximum 3 stars)** | **Total Score** | **Quality Rating^b^** |
| --- | --- | --- | --- | --- | --- | --- |
| **Cohort Studies** | | | | | | |
|  | Arnedo-Pena et al., 2015 [28] | ******** | ***** | ****** | **7** | **Good** |
|  | Gupta et al., 2016 [29] | ******** | ****** | ******* | **9** | **Good** |
|  | Owolabi et al., 2016 [31] | ******** | ***** | ****** | **7** | **Good** |
|  | Sudfeld et al., 2013 [32] | ******** | ****** | ******* | **9** | **Good** |
|  | Talat et al., 2010 [33] | ******** | ***** | ****** | **7** | **Good** |
|  | Tenforde et al., 2017 [34] | ******** | ****** | ****** | **8** | **Good** |
| **Case Control Study** | | | | | | |
|  | Mave et al., 2015 [30] | ******** | ***** | ******* | **8** | **Good** |

^a^ Wells GA, Shea B, O'Connell D, Peterson J, Welch V, Losos M, et al. The Newcastle-Ottawa Scale (NOS) for assessing the quality of nonrandomised studies in meta-analyses. Ottawa Hospital Research Institute. Available from: http://www.ohri.ca/programs/clinical_epidemiology/oxford.asp

^b^ We rated study quality by awarding stars in each domain according to the Newcastle-Ottawa Scale (NOS). Maximum possible score is 9 points, and we categorized study quality as: good (≥ 7 points), fair (5 – 6 points) and poor (< 5 points).
