# Supplementary material for: Vitamin D status and risk of incident tuberculosis disease: A nested case-control study, systematic review, and individual-participant data meta-analysis
Source: PLoS Med. 2019 Sep 11;16(9):e1002907. doi: 10.1371/journal.pmed.1002907 (PMC6738590; doi:10.1371/journal.pmed.1002907)
Supplement: S3 Table — (DOCX) [file pmed.1002907.s011.docx]

**S3 Table. Details of study identified from updated search and not included in the individual participant data meta-analysis.**

| **Study (reference)** | **Country** | **Study design and study population** | **Total Number of participants** | **Number of TB cases (%)** | **Age, years Mean (± SD)** | **Female, N (%)** | **HIV-positive cases, N (%)** | **Method of measuring vitamin D** | **Baseline 25-OH vitamin D, nmol/L mean (± SD) or median (IQR)** | **Length of follow up, years** | **TB disease definition** | **Adjusted effect estimate (95% CI) reported from study** |
| --- | --- | --- | --- | --- | --- | --- | --- | --- | --- | --- | --- | --- |
| Maceda et al., 2018^a^ | Brazil | Nested case-control study among male prisoners | 72 | 24 (33.3) | Cases 30.8 (**±** 8.9)  Controls 31.9 (**±** 9.0) | 0 (0.0) | 1 (1.4) | electrochemiluminescence immunoassay | Cases 92.5 (**±** 37.0)  Controls 93.8 (**±** 27.5) | 1.0 | Smear or culture positive | aOR for vitamin D < 75 nmol/L and microbiologically confirmed TB: 0.59 (0.13 – 2.62) |

^a^ Maceda EB, Gonçalves CCM, Andrews JR, Ko AI, Yeckel CW, Croda J. Serum vitamin D levels and risk of prevalent tuberculosis, incident tuberculosis and tuberculin skin test conversion among prisoners. Sci Rep. 2018;8(1): 997.
